# Immunohistochemical Expression of MLH1 and MSH2 in Colorectal Carcinoma and Its Correlation With Clinicopathological Parameters

**DOI:** 10.7759/cureus.105200

**Published:** 2026-03-13

**Authors:** Chokkapu Pragnya, Vijayalaxmi Patil

**Affiliations:** 1 Pathology, Shri B.M. Patil Medical College, Hospital and Research Centre, BLDE (Deemed to be University), Vijayapura, IND

**Keywords:** colorectal carcinoma, dna mismatch repair, immunohistochemistry, microsatellite instability, mlh1, mmr protein expression, msh2

## Abstract

Introduction

Colorectal carcinoma (CRC) is a major health problem worldwide and is one of the top causes of cancer deaths. Defects in DNA mismatch repair (MMR) are responsible for a few cases of CRC, and the defect mainly involves MLH1 and MSH2, which leads to microsatellite instability (MSI). Finding MMR deficiency is important for predicting outcomes, screening for hereditary conditions like Lynch syndrome, and choosing treatments such as immune checkpoint inhibitors. However, there is limited data on MMR protein expression and its clinical associations in Indian patients. The objective of this study was to evaluate the immunohistochemical expression of mismatch proteins MLH1 and MSH2 in colorectal carcinoma, and to correlate MLH1 and MSH2 expression with clinicopathological parameters such as age, gender, tumor site, histological type of tumor, and histological grade.

Materials and methods

This retrospective cross-sectional study included 45 histologically confirmed cases of CRC. Immunohistochemical staining for MLH1 and MSH2 was performed on formalin-fixed, paraffin-embedded tissue sections. Complete absence of nuclear staining in tumor cells, with intact internal controls, was interpreted as loss of expression, indicating MMR deficiency and MSI. Retained expression of both proteins was interpreted as MMR-proficient based on MLH1 and MSH2 expression, although complete assessment of microsatellite instability ideally requires evaluation of all four mismatch repair proteins (MLH1, MSH2, MSH6, and PMS2). Statistical analysis was performed to determine correlations with clinicopathological variables such as age, gender, tumor site, histological type, and histological grade.

Results

The mean patient age was 56 ± 14 years. The majority of patients (n=27, 60%) were female. The colon was the most common tumor site (n=25, 55.6%), and conventional adenocarcinoma was the main type (n=44, 97.8%). Most tumors were moderately differentiated adenocarcinomas, comprising 35 (77.8%) patients. Overall, 21 patients (46.7%) were classified as MSI (MMR-deficient), while 24 (53.3%) were MSS (MMR-proficient). MLH1 loss occurred in 19 (42.2%) patients, and MSH2 loss in nine (20%). MLH1 loss was significantly linked to patients under 50 years of age (p = 0.009). MSH2 expression did not show a significant correlation with clinical or pathological factors.

Conclusion

Many CRC cases in this study showed loss of MLH1 and/or MSH2, which suggests MMR deficiency and MSI. MLH1 loss was closely linked to early-onset CRC and may point to a hereditary risk. Regular testing for MMR proteins can help with diagnosis, screening for Lynch syndrome, and choosing the best treatment.

## Introduction

Colorectal carcinoma (CRC) is a significant global health problem and is one of the leading causes of cancer illness and death. It is the fourth most common cancer and the third most common cause of cancer death worldwide [1]. Global cancer statistics from 2020 show that CRC affected 40,408 men (6.3%) and 24,590 women (3.7%) of all cancer cases in India [2].

CRC mostly affects people over 50, but recent data indicate an increase in diagnoses among younger individuals. This increase may be linked to hereditary syndromes or early environmental exposures [3], such as a family history of CRC or exposure to certain chemicals in the environment. Certain dietary and lifestyle habits, such as a high-fat diet, low fiber intake, and sedentary behavior, can affect the risk of developing CRC. CRC risk is also influenced by genetic factors. Hereditary forms include *MUTYH*-associated polyposis, Lynch syndrome, and familial adenomatous polyposis. Other related syndromes include Peutz-Jeghers syndrome, which is characterized by distinctive skin pigmentation and gastrointestinal polyps, hereditary mixed polyposis syndrome, which involves a combination of different types of polyps, juvenile polyposis syndrome, which typically occurs in children and leads to the development of multiple polyps, and serrated polyp syndrome, which is associated with a specific type of polyp that can lead to cancer [4].

CRC develops through three main molecular pathways: chromosomal instability, CpG island methylator phenotype, and microsatellite instability (MSI) [5]. In sporadic CRC, MSI and chromosomal instability usually happen as separate pathways. MSI happens due to inherited changes in mismatch repair (MMR) genes like *MLH1*, *MSH6*, *MSH2*, *PMS2*, *EPCAM*, or *OMSR* [6].

Methods like polymerase chain reaction (PCR)-based amplification or immunohistochemistry (IHC) for MMR proteins are employed to assess MSI. PCR detects MSI, while IHC identifies the lost gene [4]. MMR genes, which serve as specific molecular markers, have been the subject of extensive research. Germline mutations cause the MMR genes to stop working, which leads to mistakes in DNA copying, changes in normal cells, and the development of tumor cells. *MSH2* and *MLH1 *are particularly significant among the MMR genes, with over 90% of deletions in them.

Although there have been advances in understanding CRC biology, there is still little information about MMR protein expression in the Indian population. Since genetic, environmental, and dietary factors can vary by population, studying MMR status is important for understanding how the disease behaves in this group. Identifying MSI is important for diagnosing, providing postoperative care, predicting outcomes, and monitoring people with CRC [7].

Aim of the study

The objective of this study was to evaluate the immunohistochemical expression of mismatch repair proteins MLH1 and MSH2 in CRC and examine how their expression relates to important clinicopathological factors such as patient age, sex, tumor location, histological type, tumor grade, and tumor stage.

## Materials and methods

This was a retrospective cross-sectional study conducted in the Department of Pathology, Shri B.M. Patil Medical College Hospital and Research Centre, Vijayapura, Karnataka, India. The study was approved by the Institutional Ethical Committee of Shri B.M. Patil Medical College, Hospital and Research Centre (reference number: BLDE(DU)/IEC-SBMPMC/123/2023-24).

Eligibility criteria

The study included 45 samples of CRC collected in the histopathology section from March 2024 to November 2025, but it did not include samples from patients who had received neoadjuvant chemotherapy or radiotherapy.

Specimens

The specimens were preserved in 10% formalin and processed routinely. Three-micron-thick sections prepared from the most suitable tissue block were deparaffinized and rehydrated using xylene and alcohol. One section was stained with hematoxylin and eosin (H&E) for morphological diagnosis. Two sections were placed on special slides that help the tissue stick, and then they were stained using specific antibodies for MLH1 (MLH1 mouse monoclonal antibody, clone ES05 (PathnSitu Biotechnologies, Secunderabad, Telangana, India)) and MSH2 (MSH2 primary mouse monoclonal antibody, clone FE11 (PathnSitu Biotechnologies)). Nuclear staining in non-cancerous cells, like stromal cells, lymphocytes, and normal colonic epithelial cells, served as a reference to show that the

IHC interpretation of MLH1 and MSH2

The specimens were examined using a light microscope. The College of American Pathologists (CAP) protocol was used, which states that any nuclear staining, including patchy staining, is considered no loss of expression. Only a complete absence of nuclear staining was considered as showing loss of expression [8]. This was evaluated by two pathologists, and there was no interobserver variability. IHC expression of MLH1 and MSH2 was correlated with prognostic factors such as the patient's age, gender, tumor site, histological type, and grade. 

In this study, we used IHC analysis for MLH1 and MSH2 only, as resources were limited. Testing just MLH1 and MSH2 can find many tumors that have MMR gene issues, but current guidelines suggest checking all four markers: MLH1, MSH2, MSH6, and PMS2 for a complete screening of MSI. As a result, cases with retained MLH1 and MSH2 expression were considered MMR-proficient based on the markers we tested, but this data does not confirm that they are definitively microsatellite stable.

Statistical analysis

The data obtained were entered in a Microsoft Excel sheet (Microsoft Corporation, Redmond, Washington, United States), and statistical analysis was performed using IBM SPSS Statistics for Windows, version 20 (IBM Corp., Armonk, New York, United States). Results were presented as mean ± SD, median, interquartile range (IQR), and frequency and percentages. Associations between categorical variables were compared using the chi-square test, and p<0.05 was considered statistically significant. 

## Results

**Figure 1:**
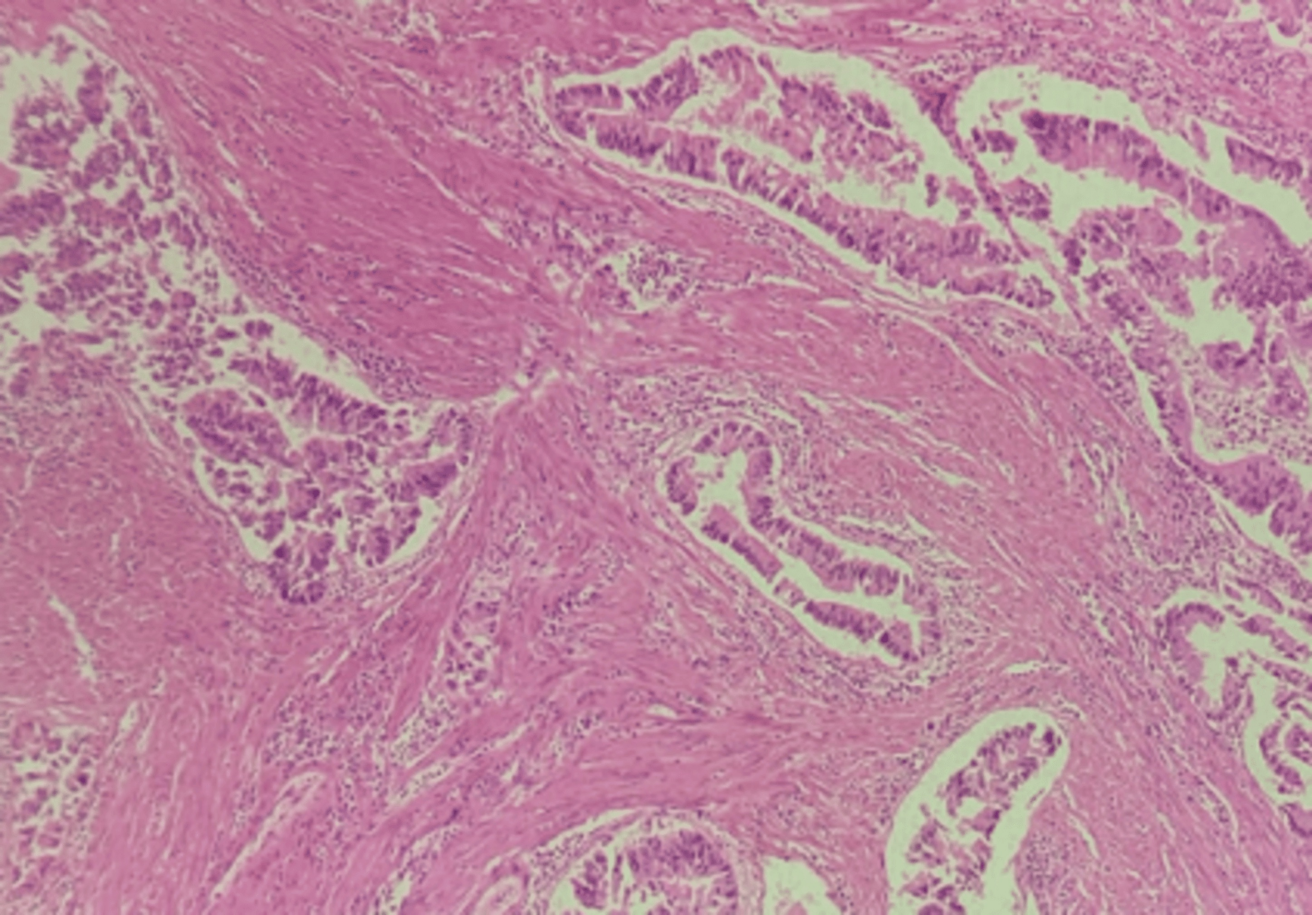
Photomicrograph of moderately differentiated adenocarcinoma (H&E, 100X) H&E: hematoxylin and eosin

**Figure 2:**
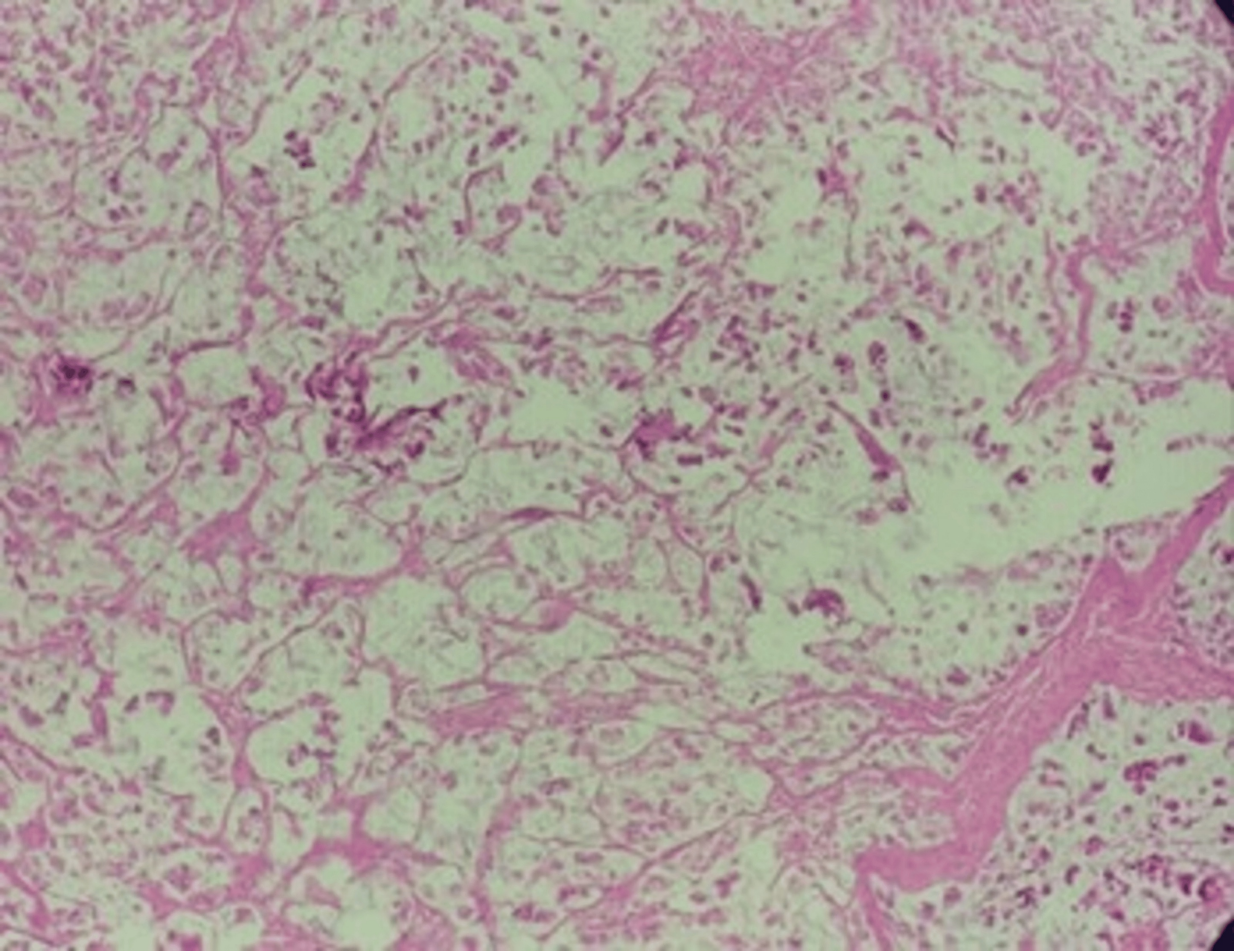
Photomicrograph of mucinous adenocarcinoma – rectum (H&E-100X) H&E: hematoxylin and eosin

A total of 45 CRC cases were evaluated. The age of patients ranged from 24 to 80 years, with a mean age of 56 ± 14 years. There was a mild female predominance, with female patients constituting 60% (n = 27) of the cases and male patients constituting 40% (n = 18), yielding a male-to-female ratio of 2:3. Tumors appeared more often in the colon (55.6%) than in the rectum (44.4%). The ascending colon was the most common site (31%), followed by the descending colon (24%). Almost all cases were adenocarcinoma not otherwise specified (NOS) at 97.8% (Figure 1), while mucinous adenocarcinoma made up 2.2% (Figure 2).

Most tumors were moderately differentiated adenocarcinomas (77.8%), with fewer being well-differentiated (13.3%) or poorly differentiated adenocarcinomas (8.9%), as shown in Table 1. Most tumors were at stage T2 (53.3%), with smaller numbers at T3 (33.3%), T4 (8.9%), and T1 (4.4%). Poorly differentiated adenocarcinomas were more likely to be at advanced stages, which was statistically significant.

**Table 1:** Association of colorectal carcinoma cases grading with staging

GRADING	T1, n (%)	T2, n (%)	T3, n (%)	T4, n (%)	Chi-square	p-value
Well-differentiated adenocarcinoma (n=6)	1 (50.0%)	4 (16.7%)	1 (6.7%)	0 (0.0%)	12.63	0.049
Moderately differentiated adenocarcinoma (n=35)	1 (50.0%)	20 (83.30%)	10 (66.70%)	4 (100%)		
Poorly differentiated adenocarcinoma (n=4)	0 (0.0%)	0 (0.0%)	4 (26.7%)	0 (0.0%)		

**Figure 3:**
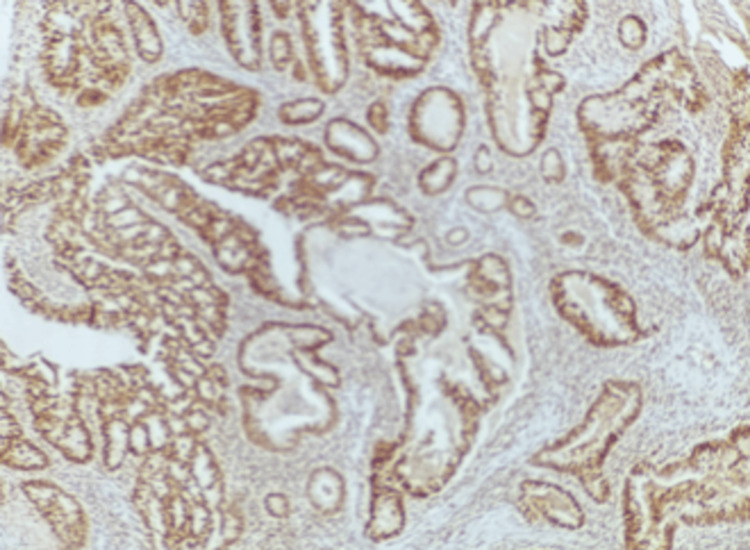
Photomicrograph showing MLH1 nuclear expression of well-differentiated colorectal carcinoma (IHC, 100X) IHC: immunohistochemistry

**Figure 4:**
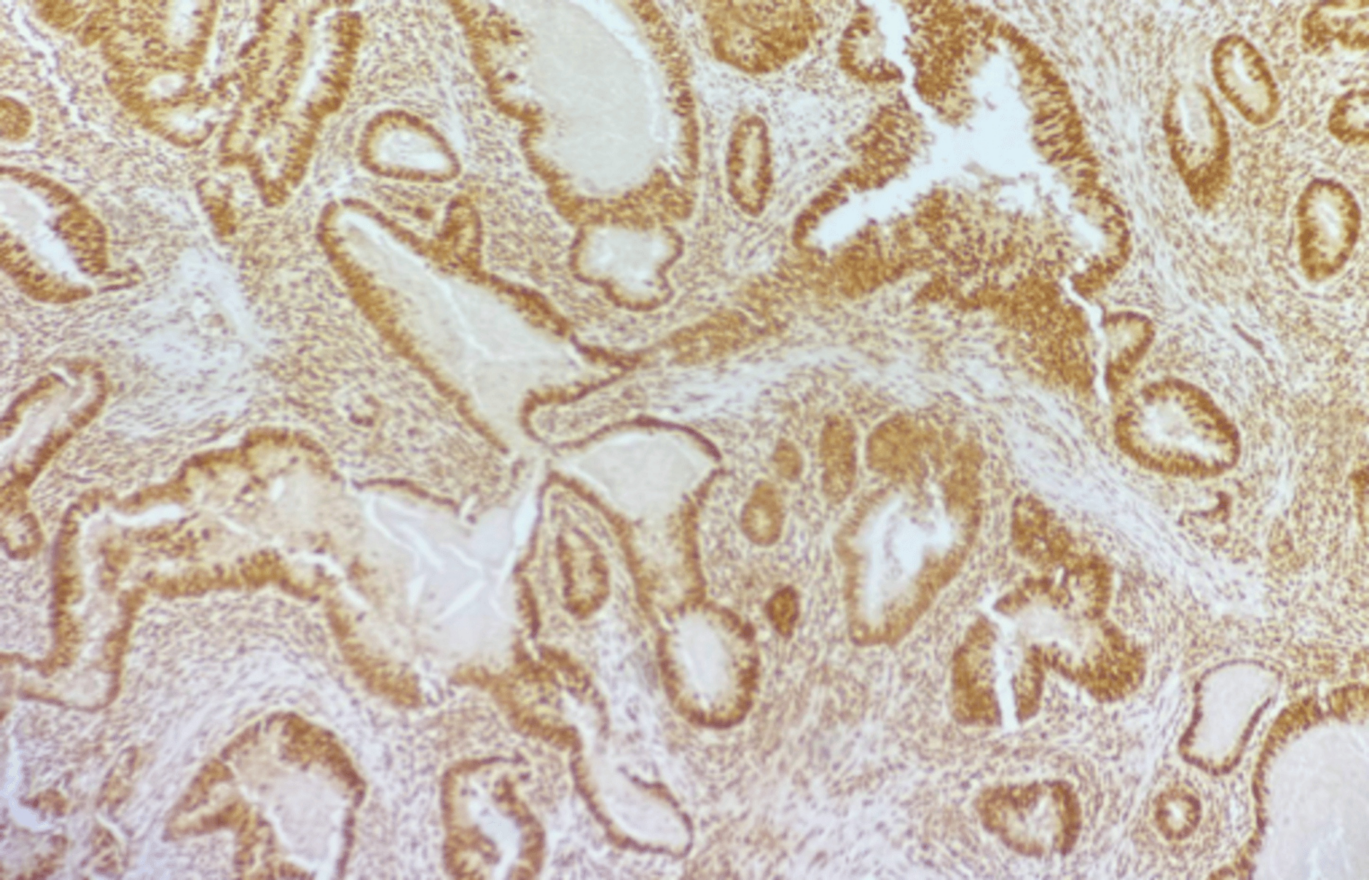
Photomicrograph showing MSH2 nuclear expression in well-differentiated colorectal carcinoma (IHC, 100X) IHC: immunohistochemistry

IHC showed that 24 cases (66.7%) had normal MLH1 and MSH2 expression. Loss of MLH1 was found in 19 cases (42.2%) (Figure 3), and loss of MSH2 in nine cases (20.0%) (Figure 4). Among tumors with loss of MLH1 expression, 77.8% also showed loss of MSH2 expression, while 33.3% retained MSH2 expression. Among MLH1-positive tumors, 22.2% showed loss of MSH2, but most (66.7%) carcinomas retained the expression. There was a significant link between MLH1 and MSH2 expression, as shown in Table 2. 

**Table 2:** Immunohistochemical expression of MLH1 and MSH2 in colorectal carcinomas cases

	MSH2 Negative (n=9), n (%)	MSH2 Positive (n=36), n (%)	Total (n=45)	Chi Square	P value
MLH1 Negative	7 (77.8%)	12 (33.3%)	19 (42.2%)	5.830	0.016
MLH1 Positive	2 (22.2%)	24 (66.7%)	26 (57.8%)		

Microsatellite status was determined based on MMR protein expression by checking the IHC expression of MLH1 and MSH2. Tumors that retained both MLH1 and MSH2 proteins were labeled as MMR-proficient using these markers. Tumors that lost one or both of these proteins were considered MMR-deficient, which may indicate MSI. Because MSH6 and PMS2 were not tested, we cannot definitively say if the tumors are microsatellite stable. Those with concurrent loss of both proteins were noted as MSI-high (MSI-H), and those with loss of either MLH1 or MSH2 as MSI-low (MSI-L). Out of 45 cases, 24 (53.3%) were MMR-proficient, 14 (31.1%) were MSI-L, and seven (15.6%) were MSI-H, as shown in Table 3. 

**Table 3:** Correlation of MMR-proficient and microsatellite instable cases with clinicopathological parameters MMR: mismatch repair; MMR-H: MMR-high; MMR-L: MMR-low; NOS: not otherwise specified

Variable	Category	MSI-H (n=7), n (%)	MSI-L (n=14), n (%)	MMR-Proficient (n=24), n (%)	Total, n (%)	Chi-square	p-value
Age (years)	≤ 50	2 (28.6%)	2 (14.3%)	13 (54.2%)	17 (37.8%)	6.282	0.043
	≥ 51	5 (71.4%)	12 (85.7%)	11 (45.8%)	28 (62.2%)		
Sex	Female	4 (57.1%)	8 (57.1%)	15 (62.5%)	27 (60.0%)	0.134	0.935
	Male	3 (42.9%)	6 (42.9%)	9 (37.5%)	18 (40.0%)		
Tumor Site	Colon	4 (57.1%)	9 (64.3%)	12 (50.0%)	25 (55.6%)	0.739	0.691
	Rectum	3 (42.9%)	5 (35.7%)	12 (50.0%)	20 (44.4%)		
Histological Type	Adenocarcinoma NOS	7 (100%)	14 (100%)	23 (95.8%)	44 (97.8%)	0.895	0.639
	Mucinous adenocarcinoma	0 (0%)	0 (0%)	1 (4.2%)	1 (2.2%)		
Histological Grade	Well-differentiated Adenocarcinoma	2 (28.6%)	0 (0%)	4 (16.7%)	6 (13.3%)	4.388	0.356
	Moderately differentiated Adenocarcinoma	4 (57.1%)	13 (92.9%)	18 (75.0%)	35 (77.8%)	-	-
	Poorly differentiated Adenocarcinoma	1 (14.3%)	1 (7.1%)	2 (8.3%)	4 (8.9%)		
Tumor Stage (T)	T1	1 (14.3%)	0 (0%)	1 (4.2)	2 (4.4%)	4.826	0.566
	T2	5 (71.4%)	7 (50%)	12 (50%)	24 (53.3%)		
	T3	1 (14.3%)	6 (42.9%)	8 (33.3%)	15 (33.3%)		
	T4	0 (0%)	1 (7.1%)	3 (12.5%)	4 (8.9%)		

## Discussion

CRC is a major health concern around the world and ranks among the leading causes of cancer-related illness and death. Although CRC was once mostly seen in older adults, it is now being found more often in younger people, especially in developing countries like India. This rise is connected to changes in lifestyle, diet, obesity, lower levels of physical activity, environmental factors, and inherited genetic risks [3]. The average age of patients in this study was 56 ± 14 years; similar findings were reported by Kheirelseid et al. [9], Mohammed and Hasan [10], Hashmi et al. [11], and Yadav et al. [4]. The higher rate of CRC in the age group of >50 years may be due to the gradual buildup of factors such as histological changes and epigenetic modifications over time [9,10].

In this study, most CRC cases were found in females, similar to the results of Lanza et al. [12] and Hashmi et al. [11]. However, Mohammed and Hasan [10], Yadav et al. [4], and Guo et al. [13] reported more cases in males. In this study, 55.6% of tumors were found in the colon and 44.4% in the rectum. Most colon tumors were found in the ascending colon. These results are similar to those of Yadav et al. [4] and Lanza et al. [12], but Mohammed and Hasan [10] found different patterns.

Adenocarcinoma (NOS) accounted for 97.8% of cases, while mucinous adenocarcinoma was seen in only one patient (2.2%). This strong dominance of adenocarcinoma matches other studies [9-13] and confirms it is the most common histopathological type of CRC worldwide. Most tumors were moderately differentiated carcinomas (77.8%), while 13.3% were well-differentiated and 8.9% were poorly differentiated. The significant link between tumor grade and stage (p = 0.049) in this study suggests that poorly differentiated carcinomas may be more advanced. This matches the findings of Hashmi et al. [11], Lanza et al. [12], and Guo et al. [13], who also reported a significant link between tumor grading and staging.

MLH1 expression was present in 57.8% of cases, while 42.2% showed loss of nuclear expression. There was a statistically significant link between MLH1 expression and patient age (p = 0.009), with loss of MLH1 more common in people under 50. This suggests a possible connection to hereditary cancer syndromes, especially Lynch syndrome, where *MLH1 *mutations often play a key role, as stated by Durhuus et al. [14]. MLH1 expression did not show significant correlations with gender, tumor site, histological type, grade, or stage. These results are in line with studies by Lanza et al. [12], Hashmi et al. [11], and Guo et al. [13]. MSH2 expression was found in 80% of cases, while 20% showed loss. Unlike MLH1, MSH2 deficiency is more often linked to germline mutations, especially in patients with Lynch syndrome, making it a useful screening marker for hereditary CRC [14]. There was no significant association between MSH2 status and age, sex, tumor site, histological type, grade, or stage. These results match the findings of Hashmi et al. [11]. However, Guo et al. [13] found links between lower MSH2 expression and both age and tumor grading. Lanza et al. [12] also reported associations between age, tumor site, and MSH2 expression. This suggests that larger sample sizes may help clarify these relationships.

A key finding in this study was a statistically significant association (p = 0.016) between MLH1 and MSH2 expression. Among tumors lacking MSH2 expression, 77.8% also showed loss of MLH1 expression, suggesting that combined loss of these MMR proteins may be more common than previously thought. The link between MLH1 and MSH2 expression is biologically reasonable because MMR proteins work as pairs, so if one protein is unstable, it can affect the other. Similar findings have been reported by Lanza et al. [12] and Wang et al. [15], highlighting the importance of testing several MMR markers at the same time.

Microsatellite analysis found that 15.6% of tumors were MSI-H, 31.1% were MSI-L, and 53.3% were MMR-proficient. Age was the only clinicopathological factor significantly related to MSI status (p = 0.043). Older patients were more likely to have MSI-H and MSI-L tumors, while younger patients were more likely to have microsatellite-stable tumors. There was no significant link between MSI status and sex, tumor site, histological type, grade, or tumor stage. These findings are consistent with Yadav et al. [4], but not consistent with the study done by Lanza et al. [12] and Guo et al. [13]

To fully evaluate mismatch repair deficiency in CRC, all four main MMR proteins, MLH1, MSH2, MSH6, and PMS2, should be tested. This study only tested MLH1 and MSH2. Even though it's usual for CRCs with MMR deficiency to lose MLH1 and MSH2, some tumors might only lose MSH6 or PMS2 and still have microsatellite instability, even if MLH1 and Because of this, normal results for MLH1 and MSH2 should be interpreted with caution. Testing all four proteins is recommended to accurately detect microsatellite instability and Lynch syndrome.

Strength of the study

This study presents new data on MLH1 and MSH2 expression in CRC among an Indian population, where there is limited research. Our findings show significant links between MLH1 loss and younger age, as well as between MLH1 and MSH2 expression. These results provide useful insight into the molecular features of CRC in this group. Including MLH1 and MSH2 testing in standard diagnostic protocols can improve prognosis, guide personalized treatment, and support early detection of hereditary CRC syndromes.

Limitations of the study

This study’s small sample size (n = 45) and single-center design make it challenging to apply the findings more broadly. This study only used IHC analysis for MLH1 and MSH2 due to resource limitations, even though current guidelines recommend testing the full MMR panel, which includes MLH1, MSH2, MSH6, and PMS2. Because of this, tumors with loss of MSH6 or PMS2 might have been missed, and some cases with normal MLH1 and MSH2 could still be MMR-deficient or related to Lynch syndrome. Also, MSI status was estimated using IHC instead of being confirmed by PCR-based MSI testing. Future research that uses the full MMR panel and molecular testing would give a more complete assessment. We also could not collect follow-up or survival data and assess the long-term impact of MMR status.

## Conclusions

In our study, most CRCs were moderately differentiated adenocarcinomas, and there was a significant association between tumor grade and stage. To fully evaluate MMR deficiency in CRC, all four main MMR proteins, MLH1, MSH2, MSH6, and PMS2, should be tested, while this study only tested MLH1 and MSH2. We found MLH1 loss of expression in 42.2% of CRC cases, and MSH2 loss of expression in 20% of the CRC cases, and a significant association was found between loss of MLH1 and MSH2. MSI appeared in more than one-third of tumors, with age as the only clinicopathological factor significantly linked to MSI status. These results support using MLH1 and MSH2 IHC routinely in CRC to improve prognosis and help identify tumors with MMR deficiency.
